# *Grewia asiatica* Berry Juice Diminishes Anxiety, Depression, and Scopolamine-Induced Learning and Memory Impairment in Behavioral Experimental Animal Models

**DOI:** 10.3389/fnut.2020.587367

**Published:** 2021-01-15

**Authors:** Imran Imran, Sana Javaid, Aroosa Waheed, Muhammad Fawad Rasool, Abdul Majeed, Noreen Samad, Hamid Saeed, Faleh Alqahtani, Mohammed M. Ahmed, Faten Abdullah Alaqil

**Affiliations:** ^1^Department of Pharmacology, Faculty of Pharmacy, Bahauddin Zakariya University, Multan, Pakistan; ^2^Department of Pharmacy, The Women University, Multan, Pakistan; ^3^Department of Pharmacy Practice, Faculty of Pharmacy, Bahauddin Zakariya University, Multan, Pakistan; ^4^Department of Biochemistry, Faculty of Science, Bahauddin Zakariya University, Multan, Pakistan; ^5^Section of Pharmaceutics, University College of Pharmacy, University of the Punjab, Lahore, Pakistan; ^6^Department of Pharmacology and Toxicology, College of Pharmacy, King Saud University, Riyadh, Saudi Arabia

**Keywords:** *Grewia asiatica*, amnesia, anxiety, depression, Morris water maze, escape latency

## Abstract

*Grewia asiatica* L. fruit natively called phalsa is a popular berry of Pakistan and widely consumed in the form of fresh juices and carbonated drinks in the summer season. The berry is enriched with antioxidants such as phenols, flavonoids, anthocyanins, and vitamin C. Scientifically, it is the least explored berry in terms of neuromodulatory activities, and therefore, in the designed study, chronically fed rats with the different dilutions (5%−30%) of fruit juice were subjected to behavioral assessment for anxiety, depression, and cognition (spatial memory) followed by biochemical analysis of isolated brains. Results revealed a prominent impact of 20 and 30% dilutions of fruit exudate as treated animals showed anxiolytic behavior to central zone (*P* < 0.05) of open field test (OFT) and open arms of elevated plus maze (EPM) (*P* < 0.05) in anxiety models. Overall, immobility of rats treated with a higher concentration of exudate in forced swim test (FST) was reduced (*P* < 0.05) presenting antidepressant-like activity. Moreover, in learning and memory experimental models, the treated animals reversed scopolamine-induced amnesic effects as evident from improved step-through latencies (*P* < 0.05 vs. scopolamine; passive avoidance test), spontaneous alternation behavior (*P* < 0.05 vs. scopolamine; Y-maze test), discrimination index (*P* < 0.05 vs. scopolamine; novel object recognition test), and escape latencies (*P* < 0.05 vs. scopolamine; Morris water maze). Biochemical studies of isolated brains from treated rats demonstrated significantly elevated levels of superoxide dismutase and glutathione peroxidase (*P* < 0.05), whereas levels of acetylcholinesterase and malondialdehyde level (*P* < 0.05) were reduced, indicating its potential to reduce oxidative damage in the brain and modulation with the cholinergic system. The outcomes of studies support the benefits of phytoconstituents possessed by *G. asiatica* fruit in the amelioration of neurological disorders that could be due to their antioxidative capacity or due to interaction with GABAergic, serotonergic, and cholinergic systems in the brain.

## Introduction

It is well-known that healthy nutrition is an important key to healthy living. The therapeutic importance of nutritional substances has been accepted for years. A recent survey reports that 33% of Americans tend to include those components in their daily diet that prove beneficial for their health as well (1). Hippocrates' philosophy also claims that interventions made in a patient's nutrition can help him recover from ailment (2). Fruits include various nutrients that help promote health and protect against diseases (3). When taken as a whole, they work best due to the synergistic effect of all of their unique constituents. The presence of several bioactive constituents in fruits tends to boost the overall health of consumers. That is why the fruit is designated as one of the most valuable nutritional sources rich in fiber, vitamins, carbohydrates, minerals, and antioxidants, which are crucial for a healthy lifestyle (4).

*Grewia* is the only genus of “*Tiliaceae*” that yields edible fruit and consists of almost 150 species scattered throughout the world (5). Out of these, about 10 species are found in Pakistan (6). *Grewia asiatica* L. is one variety that grows wild and is cultivated as well, providing a sour, acidic fruit used in South Asian countries (7). The fruit of this plant is locally known as phalsa. This small, purplish, and sour fruit is a seasonal gift of nature and becomes excessively available in District Multan, Pakistan, from late April or early May and is available until June. The fruit is commonly used to make juice, squash, and carbonated drinks, which are used as healthiest beverages in the summer due to their soothing effect (6).

*Grewia asiatica* L. not only is the source of a delicious edible fruit but also possesses a variety of medicinal benefits as its fruit holds anti-inflammatory, antimicrobial, anticancer, and antidiabetic characteristics (8). Its fruit owns nutritionally significant amino acids, i.e., threonine and methionine (6) and various vitamins (6). Chemical analysis of seeds reports the presence of oil comprising palmitic, stearic, oleic, and linoleic acids (9). Analysis of the presence of micronutrients reveals that iron is found in the highest concentration (10). Besides these, various compounds and secondary metabolites are reported in fruits of *G. asiatica*, i.e., naringenin-7-*O*-β-D-glucoside, pelargonidin 3,5-diglucoside, cyanidin-3-glucoside quercetin, quercetin 3-*O*-β-D-glucoside, catechins, and tannins (11). When evaluated for anthocyanin content, the occurrence of cyanidin 3-O-(6”-acetyl-glucoside), peonidin-3-O-glucoside, and pelargonidin 3-O-(6”-acetyl-glucoside) was exposed, which also impart pharmacological benefits to this fruit (12).

Fruits of the various plants have demonstrated potential in resolving brain-related disorders (13–15), so the current need is to evaluate the fruit of local plant *G. asiatica* to cure neurological diseases that have become much more prevalent in Pakistan (16). There are more than 600 types of neurological disorders (17), but the most prevalent ones are dementia, Alzheimer's disease, epilepsy, schizophrenia, and Parkinson's disease (18). Symptoms of almost all these neurological disorders are structural, functional, or biochemical abnormalities, leading to frequently prominent consequences such as loss of coordination, seizures, confusion, and reduced cognition (19). Challenges in treating neurological disorders include high costs (20) and low tolerability of therapeutic drugs due to related side effects (21). These limitations provide researchers the impetus to discover alternative options to provide improved medication adherence and remedial outcomes in patients. According to WHO, more than 70% of the population of developed countries still rely on natural remedies due to their safety, cost-effectivity, and easy availability (22). It has also been reported that the youth believe that choosing food wisely will have a positive therapeutic effect on their lives (1).

The current investigational study has been planned to evaluate the impact of the freshly made dilutions of *G. asiatica* fruit (phalsa) juice on learning, anxiety, and depression-like behavior by employing different animal behavioral models. Additionally, biochemical evaluation of brain activity was performed through a series of experiments. We expected to scientifically prove for the first time the ameliorative potential of this indigenous plant berry on brain functions.

## Materials and Methods

### Fruit Collection and Preparation of Fresh Concentrate

The fresh mature fruit of *G. asiatica* L. was purchased locally (15 kg) in May from Multan, Pakistan. After authentication through an expert botanist Dr. Zafar Ullah Zafar (associate professor, Institute of Pure and Applied Biology, BZU Multan), voucher no. R. R. Stewart 473 was submitted into the herbarium. The fruit was thoroughly washed with water to remove impurities and blended in water (1 kg fruit berries:0.5 L water) using a commonly used food blender to make a concentrated fruit juice (exudate). Freshly prepared juice was first filtered through a sieve to remove seed debris and then stored into non-reacting glass bottles at 4°C in the refrigerator. Then, 5, 10, 20, and 30% dilutions were made by mixing prepared exudate with water.

### Animals

Sprague-Dawley (SD) male rats (150–250 g) were included in the current study. Animals were purchased from the National Institute of Health, Islamabad, and housed in the animal house (Faculty of Pharmacy, Bahauddin Zakariya University, Multan) under strictly regulated maintenance conditions (25°C, 12-h light/dark cycle; lights on 8:00 am) and provided with standardized rodent chow to eat. Animals were adapted to experimentation sites 60 min before the test. All behavioral experiments were carried out from 8:00 a.m. to 6:00 p.m. The intensity of aversive stimuli was kept at a minimum in behavioral tests to avoid any carryover effects of one test on to the next. All animal studies were performed after availing authorization from the Department of Pharmacology Ethical Committee BZU, Multan (EC/12-PHL-2017 dated 22-05-2017) and were accomplished by following instructions of the Institute of Laboratory Animal Resources (ILAR), Commission on Life Sciences, National Research Council (NRC, 1996).

### Drugs and Chemicals

Piracetam, chloral hydrate, scopolamine, 5,5-dithio-bis-(2-nitrobenzoic acid) (DTNB), 2-thiobarbituric acid (TBA), and acetylthiocholine iodide were purchased from Sigma-Aldrich, USA. Diazepam and fluoxetine were procured from Roche Pharma (Switzerland) and Hilton Pharma (Pakistan), respectively. Isoflurane (Forane® Abbots Laboratories, USA) was a kind gift by Ahsan Medicine Agency, Multan, intended for the purpose of induction of anesthesia. All drugs and chemicals used in studies were highly purified and of research-grade.

### Animal Groupings and Treatments

**Figure 1:**
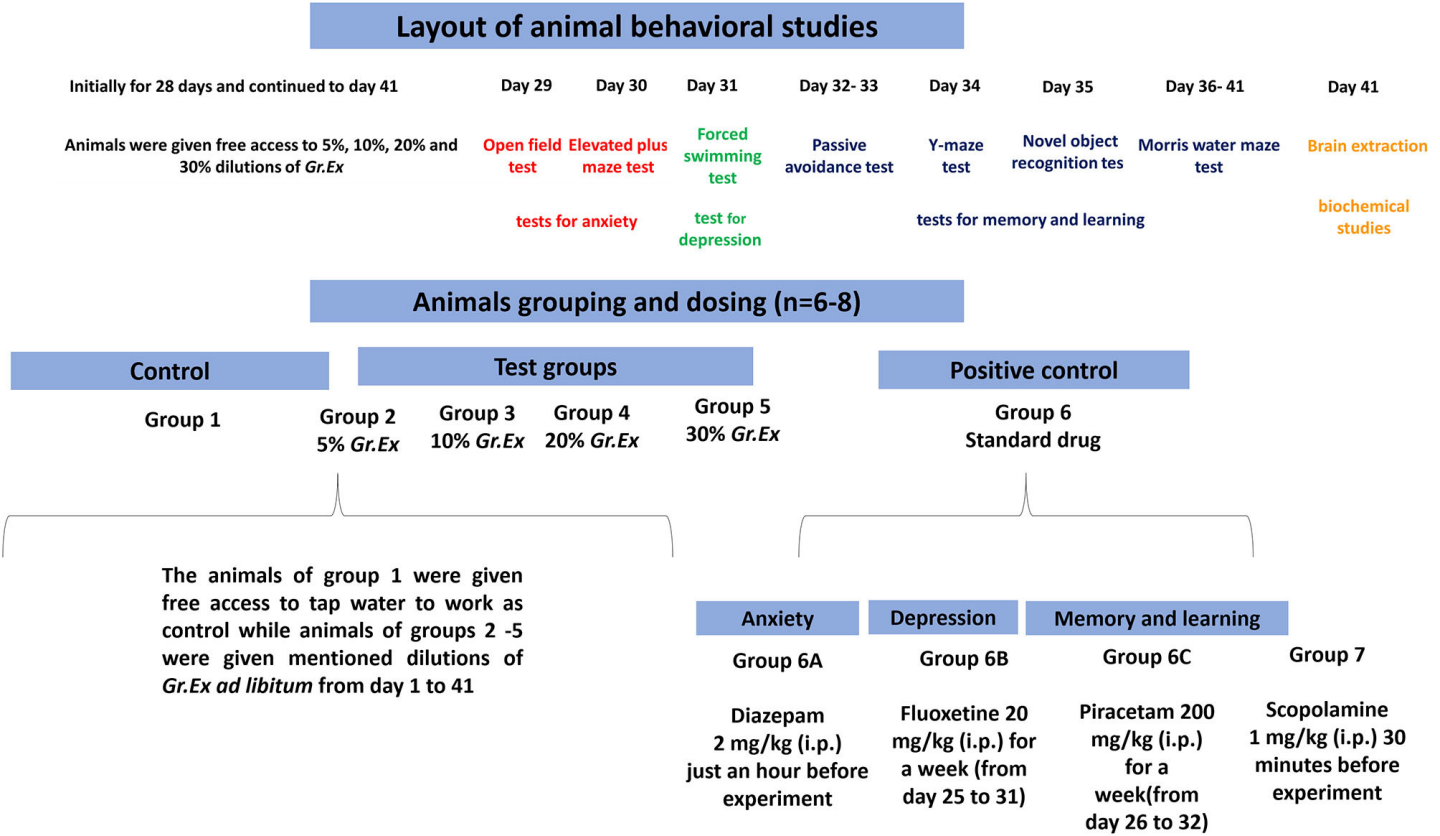
Schematic presentation of behavioral studies.

Randomly selected animals were divided into nine different groups (6–8 animals/group). Group 1 was considered as a control group and simply fed on tap water throughout the period of the study. Rats of groups 2 to 5 were administered *ad libitum* 5, 10, 20, and 30% dilutions (v/v in tap water) of *G. asiatica* exudate (*Gr.Ex*) for 28 days *via* standardized water bottles placed in the cages. After 28 days of consumption of juice, the behavioral studies were initiated, but animals of groups 2–5 were given free access to mentioned dilutions of *Gr.Ex* until day 41 of the experiment (Figure 1). In all behavioral experiments, the dilutions of *Gr.Ex* were withdrawn an hour before the commencement of the experiment.

During the behavioral test for anxiolytic activity, diazepam [2 mg/kg, intraperitoneally (i.p.)] was administered acutely 30–60 min before to one positive control group 6A (*n* = 6). Fluoxetine at the dose of 20 mg/kg i.p. was given once a day for 1 week (days 25–31) to one separate group of rats designated as group 6B (*n* = 6) to work as a positive control in the forced swimming test. For studies affiliated with the learning and memory tasks, we introduced two new separate groups of rats such as group 6C and group 7. Group 6C was a positive control to which piracetam 200 mg/kg was administered i.p. once daily for 1 week from days 26 to 32. Scopolamine (1 mg/kg, i.p.) was acutely administered 30 min before to animals of Groups 2–5, 6C, and 7. The day-wise scheme of designed experiments and animal grouping is summarized in Figure 1.

### Methanol Extract of *Grewia asiatica*

Here, 500 mL of freshly prepared 5, 10, 20, and 30% *G. asiatica* fruit juice was individually mixed with 500 mL of methanol (100%) for 3–4 days at 25°C. The mixture was shaken intermittently to allow maximum extraction of active constituents. After this, the procedures of filtration and successive evaporation were carried out. The extracts were obtained with a yield of 0.62, 1.37, 2.69, and 4.05% for 5, 10, 20, and 30% dilutions of fruit pulp, respectively. The prepared extracts were kept at a minimal temperature in the refrigerator until further experimentation.

#### Estimation of Total Phenolic Content

*Gr.Ex* was evaluated for phenolic compounds by adopting the Folin–Ciocalteu method (23). For assessment, 200 μl of each methanolic extract (1 mg/ml) was increased to 3 mL by using distilled water to which 0.5 mL of Folin–Ciocalteu reagent (50% v/v) was inoculated. After 3 min, 2 mL of 20% sodium bicarbonate (w/v) was introduced, and the resulting solution was reserved in the dark for an hour. The absorbance of this mixture was checked at 650 nm, and phenolic content was computed through the calibration curve. Outcomes had been articulated as mg of gallic acid equivalent (mg GAE) per gram of dry weight.

#### Estimation of Total Flavonoid Content

The aluminum chloride method was employed during this testing (24). In brief, 1 mL of test extract (1 mg/ml) was mingled with 4 mL of water followed by the addition of 0.3 mL of sodium nitrate (5%), and the components were allowed to mix for 5 min. Next, 0.3 mL of aluminum chloride (10%) was added, and the mixture was allowed to incubate at room temperature for 6 min. Afterward, 1 mL of sodium hydroxide (1 M) was inoculated and the volume of the mixture was made 10 mL by using distilled water. Absorbance was measured at 510 nm. The entire testing was conducted thrice using quercetin as a standard, and conclusions were represented as quercetin equivalent per (mg QE) gram of dry weight (24).

#### Estimation of Total Anthocyanin Content

The anthocyanin content was evaluated by taking 1 mL of extract (1 mg/ml) to the flask to prepare two dilutions (25). One dilution was made with potassium chloride buffer (pH 1.0) and the other with sodium acetate buffer (pH 4.5), and both solutions were allowed to equilibrate for 15 min. The absorbance of each dilution was noted at 510 and 700 nm using blank distilled water as a control.

The absorbance of the diluted sample was calculated using the formula:

$$\begin{array}{l} A=\left[\left(A510-A700\right)pH1.0-\left(A510-A700\right)pH4.5\right] \end{array}$$

While anthocyanin content was figured out through the given formula:

$$\begin{array}{l} Anthocyanin\;content\;\left(mg/ml\right)=\left[\left(A\;\times \;MW\;\times \;DF\;\times \;1,000\right)/\varepsilon \times 1\right] \end{array}$$

where MW denotes molecular weight, DF is the dilution factor, and ε is the molar absorptivity.

#### Estimation of Antioxidant Potential

The plant's antioxidant capacity was assessed through 1,1-diphenyl-2-picrylhydrazyl (DPPH) and 2,2′-azino-bis-(3-ethylbenzothiazoline-6-sulfonic acid) (ABTS) assays.

##### 1,1-Diphenyl-2-Picrylhydrazyl Method

*Grewia asiatica* was evaluated for antioxidant potential by adding 1 mL of methanolic DPPH (0.1 mM) to 3 mL of different concentrations (5–30 μg/ml) of each methanolic extract under study (26). The solution was mixed by vigorous shaking and kept at 25°C for 0.5 h. After that, absorbance was noted at 517 nm. Whole experiments were conducted in *n* = 6 while using ascorbic acid as the standard. The outcomes were indicated as IC_50_, while % DPPH inhibition was computed by the given equation:

$$\begin{array}{l} \%\text{ DPPH inhibition}=\left[{\text{A}}_{0}\;-\;{\text{A}}_{1}/{\text{A}}_{0}\right]\;\times \;100 \end{array}$$

where A_0_ is the absorbance of only methanol (control), and A_1_ is the absorbance in the presence of test or standard sample.

##### 2,2′-Azino-Bis-(3-Ethylbenzothiazoline-6-Sulfonic Acid Method

The radical hunting potential of *G. asiatica* fruit pulp was estimated through the method described (27). For this assay, ABTS^+^ was freshly prepared by combining 7 mM of ABTS solution with 2.45 mM potassium persulfate. This blend was maintained in the dark for 16 h. Later, this solution was diluted with methanol until the absorbance reaches 0.700 at 734 nm. Afterward, 3.995 mL of this ABTS^+^ solution was added to 5 μl of methanolic extract and mixed thoroughly. After 0.5 h, absorbance was recorded at 734 nm. All experimentation was repeated thrice while using the Trolox as standard and IC_50_ was expressed. In addition, % scavenging effect was determined through the given calculation:

$$\begin{array}{l} \%\text{ inhibition}=\left[\left(\text{AB }-\text{ AA}\right)/\text{AB}\right]\;\times \;100 \end{array}$$

where AB is the absorbance of only methanol (control), and AA is the absorbance in the presence of test compound or standard.

#### Estimation of Acetylcholinesterase Inhibitory Activity

The anti-cholinesterase capability of *Gr.Ex* was evaluated by the method described (28). For the assay, 100 μl of DTNB (3 mM), 40 mL of tris buffer (50 mM, pH 8.0), and 20 μl of acetylcholinesterase (AChE) (0.26 U/ml) were added to 96-well plates. Later, 20 μl of test extract in different concentrations (25–500 μg/ml) solubilized in a buffer comprising not more than 10% methanol were added to these wells. After allowing the incubation for 0.25 h, the absorbance was analyzed at 412 nm to consider as blank. To initiate the enzymatic reaction, 20 μl of acetylthiocholine was added and absorbance was checked after 5 min for 20 min. All experiments were repeated thrice using physostigmine as a standard, and outcomes were expressed as % inhibition (29, 30) calculated by the formula:

$$\begin{array}{l} \%\text{ inhibition}=\left[\Delta \text{A }-\;\Delta \text{B}/\Delta \text{A }\times \;100\right] \end{array}$$

where ΔA is the absorbance of the blank, and ΔB is the absorbance of the sample.

### Behavioral Studies for Anxiolytic Activity

#### Open Field Test

The open field test (OFT) utilizes the innate exploratory behavior of rats to evaluate anxiety, as they are naturally frightened of lighted areas but tend to explore them as well (31, 32). The test apparatus consists of a poly-acrylate-made square-shaped field (80 × 80 cm) having walls high enough (40 cm) to avoid animal escape during the test. Each animal was individually placed in the arena of the open field. Parameters such as the number of entries in the central zone and time spent there were monitored for 5 min and subsequently analyzed by ANY-maze software (V 6.1, Stoelting Co., USA). The arena was cleaned thoroughly and wiped with 70% isopropyl alcohol to remove any residual smell of the previous session.

#### Elevated Plus Maze Test

The elevated plus maze test (EPM) is a dependable and trustworthy test employed by scientists (33, 34) to evaluate anxiolytic behavior in rodents. The test is conducted in a 50-cm-high plus (+)-shaped apparatus (110 × 10 cm) having bare and walled arms. Rats naturally prefer to stay in closed areas but attempt to explore open zones at the same time. On test day, each animal was positioned to face exposed and unprotected arms to allow the exploration of the whole maze for 5 min. The open and closed arm visits, as well as time spent there, were observed by ANY-maze software (V 6.1, Stoelting Co., USA).

### Behavioral Test for Depression

#### Forced Swimming Test

The forced swimming test (FST) is based on the rodent's reaction to the threat of drowning, and the resulting response is used to estimate their vulnerability to depression-like behavior (35). The animals are subjected to forced swimming in a deep acrylic glass tank (34.5 cm × 23.5 cm) filled with water to the level that they can easily float without touching their paws to the bottom of the tank. Rats were individually observed for a duration of 5 min for their movements and immobility, and these parameters were evaluated with the help of behavioral assessment software ANY-maze V 6.1, Stoelting Co., USA) to assess the antidepressant-like potential of *Gr.Ex*.

### Behavioral Tests for Memory and Learning

#### Step-Through Passive Avoidance Test

The step-through passive avoidance test (ST-PAT) uses the conceptualization of placing rodents into the aversive bright zone from which they try to escape into an innately preferred dark environment where an unpleasant stimulus is delivered (36, 37). The test was conducted in an apparatus having two compartments of the same dimensions (20 cm × 20 cm × 20 cm) interconnected by an opening (10 cm × 10 cm). One compartment was illuminated with a bulb (100 W; halogen lamp) as a light source, and the other was a dark chamber having stainless steel floor to provide electric foot shock. The sequence of ST-PAT was divided into three segments, i.e., (1) training phase, (2) short-term memory assessed after 1 h of shock, and (3) memory retrieval assessed after 24 h of shock. In the training phase of 5 min, all animals received an electric shock of 0.5 mA for 1 s (Coulbourn Instruments Animal Shocker, USA) through the stainless-steel floor to all animals. Subsequent to the training phase, the animals of different groups (2–5, 6C, and 7) were given scopolamine (1 mg/kg, i.p.) 30 min before the judgment of memory associated with aversive stimuli (1 h post-shock session). The same procedure for the memory retention was repeated with all animals after 24 h of shock but without the amnesic drug. Total step-through latencies and duration in the dark compartment in 1 and 24 h post-shock sessions were noted for the duration of 5 min.

#### Y-Maze Test (Spontaneous Alternation)

This memory test was conducted in a trio-arm (A, B, and C) maze (55 cm × 10 cm × 15 cm) arranged adjacently at 120° (38, 39). After 30 min of scopolamine administration, rats were placed in any arm of the maze and allowed to explore spontaneously. Entries in each arm with a sequence of arm entries (ABC, BCA, CBA, etc.) were observed for 5 min to estimate their spontaneous alternation behavior. Animals showing increased alternation behavior indicated enhanced spatial memory.

% Spontaneous alternation was determined through the formula:

$$\begin{array}{l} \%\text{Spontaneous alternation }(\%\text{SAP})=[(\text{Number of alternations})/\\ (\text{Total arm entries}-\text{2})]\times 100. \end{array}$$

#### Novel Object Recognition Test

The novel object recognition (NOR) test operates on rodent's instincts to discover new surroundings for memory assessment (40, 41). The test apparatus comprises an open box (80 cm × 80 cm × 40 cm) in which animals were placed for two consecutive sessions, i.e., one comprising two identical objects and another session in which one old object was replaced by a new one. Animals were initially acclimatized in the behavioral room for at least 60 min. Subsequently, rats in the open field were exposed with familiar objects for the duration of 5 min. The animals were permitted to investigate these similar objects through sniffing or touching at a short distance (<2 cm). After 1 h, a new object was presented in the cage replacing one of the previously explored objects, and animal behavior was recorded for 5 min. The time taken by animals to explore the familiar and new object was noted to calculate the discrimination index.

$$\begin{array}{l} \text{Discrimination index}=[(\text{Time with novel object}\\ -\text{ Time with familiar object})÷(\text{Time with novel object}\\ +\text{ Time with familiar object})] \end{array}$$

#### Morris Water Maze Test

The Morris water maze (MWM) test is employed to estimate memory and spatial learning in rodents (42–44). The test apparatus comprises a spherical tub-like container (150 cm diameter and 50 cm height) having a smooth internal surface. Water with some opacifying agent (milk or non-toxic dye) was filled in this tank up to 32 cm high, and a square platform (10 × 10 cm) made of plastic was placed 2 cm below the water surface. Various distinct geometrical signs (▴, □, ◦, **—**) were displayed on the inner side of the water tank (proximal cues) and simultaneously mounted on the stands in the surroundings of the tank (distal cues) to provide spatial reference points to the animals (44, 45). Initial training trials (training days) of animals were carried out without amnesic drug for the first 2 days (four trails/animal/day of 2-min duration) by placing a platform in the southwest (SW) quadrant of the tank. During the training session, if the animal could not locate the platform within 120 s, then the rats were slightly pushed toward the platform and made them stay on it for 10 s for the formation of spatial memory. For the next 3 days (experimental trials), the rats were individually tested to locate the hidden platform for 2 min in scopolamine-induced amnesic condition (escape latencies). After 24 h of completion of the experimental trial, animals were tested in the water maze without platform (probe day), and entries in the targeted platform quadrant, swimming time, and distance traveled were observed to assess reference memory associated with the previous positioning of the platform. Scopolamine in the experimental and probe sessions was administered 30 min before to the animals of groups 2–5, 6C, and 7. *Ad libitum Gr.Ex* dilutions (5, 10, 20, and 30%) were withdrawn 1 h before the start of any session of the MWM test. The recorded videos were subsequently accessed with the help of behavioral tracking software, i.e., ANY-maze version 6.1, Stoelting Co., USA.

### Biochemical Assessment of Brain Activity

For biochemical assays, animals were deeply anesthetized in an anesthesia induction chamber (World Precision Instruments, USA) by the inflow of 5% isoflurane v/v mixed with synthetic air (Lab-Gas, Multan, Pakistan) by Rodent Anesthesia Vaporizing System (Kent Scientific Corporation USA). The flow of isoflurane vapors was kept constant for 1–2 min until animals were deeply anesthetized with loss of all reflexes and subsequently decapitated by rodent guillotine to isolate brains.

### Brain Homogenate Preparation

Animals were randomly selected from all treatment groups immediately at the end of the MWM task. They were sedated with isoflurane and decapitated for brain extraction. Isolated brains were appropriately stored in normal saline at 8°C. For brain homogenate preparation, 0.3 g of the brain sample was centrifuged at 12,000 g with 3 mL of phosphate buffer (pH 7.4) for 20 min at low temperature (46). The obtained homogenate was further assayed to investigate the level of AChE, malondialdehyde (MDA), superoxide dismutase (SOD), and glutathione peroxidase (GPx) in the animal brain.

#### Acetylcholinesterase Activity

Brain homogenate was assayed for AChE activity according to the method described (28). Brain homogenate (0.4 ml) was combined with DTNB (100 μl) and 0.1 M phosphate buffer having pH 8 (2.6 ml). The absorbance of this mixture was noted at 412 and taken as control. Later, ATC (5.2 μl) was added to the cuvette, and the change in absorbance was noted for 10 min.

#### Malondialdehyde Assay

After minor modifications in the procedure prescribed (47), 2 mL mixture of 15% trichloroacetic acid (TCA) and 0.38% thiobarbituric acid (TBA) were mixed with 100–500 μl of the homogenized brain. The resulting combination was thoroughly mixed and boiled on a water bath for 15–20 min. This mixture was then chilled at 4°C, followed by subsequent centrifugation (3,500 rpm) for 10–15 min. The absorbance of the supernatant obtained was checked at 532 nm.

#### Superoxide Dismutase Assay

This assay was performed by following the procedure described (48). Based upon the conversion of nitroblue tetrazolium (NBT) to insoluble formazan, 0.5 mL of the homogenized brain was mixed with a solution containing 0.4 mL of NBT (24 μM), 1 mL of sodium bicarbonate (50 mM), and 0.2 mL of ethylenediaminetetraacetic acid (EDTA) (0.1 mM). Later, 0.4 mL of hydroxylamine hydrochloride (1 mM) was inoculated to initiate the reaction, and absorbance was monitored for 5 min at 560 nm.

#### Glutathione Peroxidase Assay

The test was performed by following the illustrated method (46). Here, 0.3 mL of brain homogenate was combined with 0.1 mL of sodium azide (10 mM), 0.1 mL of H_2_O_2_ (1 mM), 0.3 mL phosphate buffer (pH 7.4, 0.1 M), and 0.2 mL of reduced glutathione (2 mM). The resultant blend was incubated for 0.25 h at 37°C, and 0.5 mL of 5% TCA was added to stop the reaction. Centrifugation was done for 5 min at 1,500 g, and separated supernatant was removed. Phosphate buffer (0.2 ml) and DTNB (0.7 ml) were reacted with supernatant obtained (0.1 ml) to note the absorbance at 420 nm.

### Statistical Analysis

The data were expressed as mean ± SEM (*n* = 6 for behavioral studies and *n* = 3 for biochemical studies). The data normality was checked through the Kolmogorov–Smirnov test. Analysis of most of the parameters of the behavioral and biochemical investigation was done through one-way ANOVA with Tukey's *post hoc* test. However, latencies checked in PAT and MWM and duration in the dark in PAT were evaluated by two-way ANOVA and Tukey's *post hoc* test.

## Results

### Total Phenolic, Flavonoid, and Anthocyanin Contents

**Figure 2:**
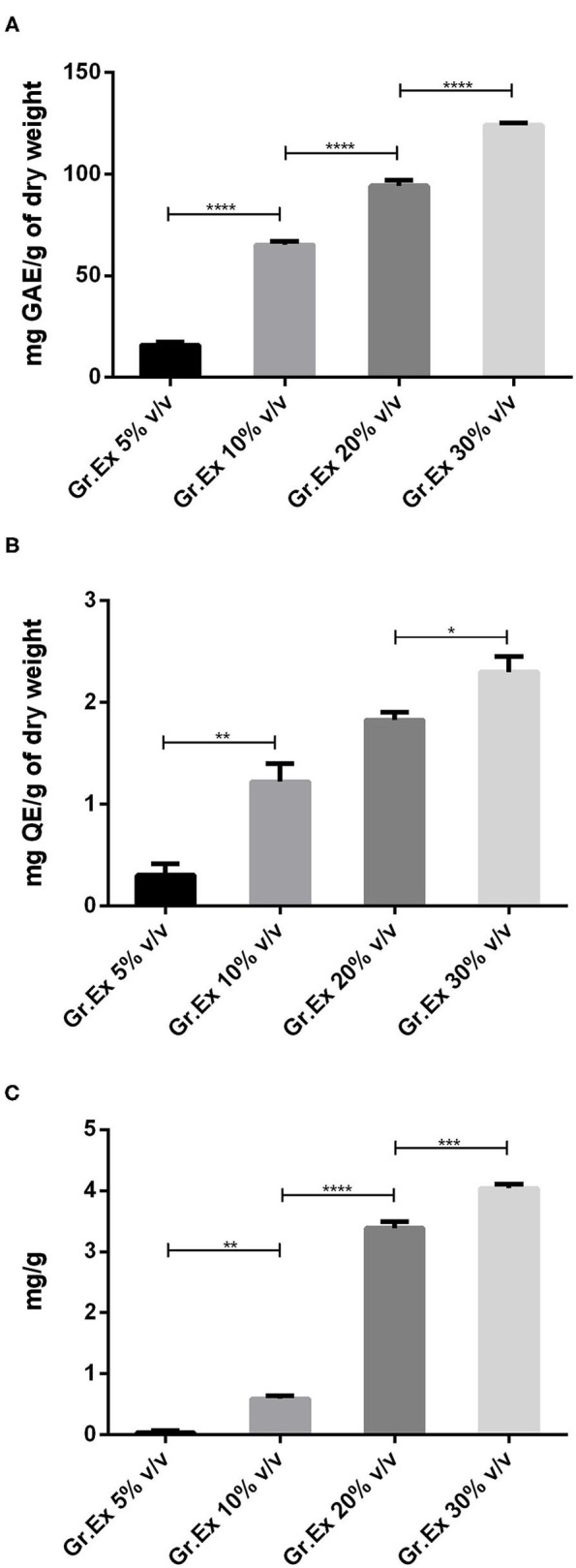
Assessment of total phenol **(A)**, flavonoid **(B)**, and anthocyanin **(C)** content in methanolic extracts of different concentrations of fruit pulp of *Grewia asiatica*. Data expressed as mean ± SEM (*n* = 3) and evaluated by one-way ANOVA followed by Tukey's test. *P < 0.05, **P < 0.01, ***P < 0.001, ****P < 0.0001, comparison between 5 and 10%, 10 and 20%, and 20 and 30% dilutions of *G. asiatica* exudate (*Gr.Ex*).

When methanolic extracts prepared from the different concentrations of fruit pulp were evaluated for the presence of phytoconstituents, the results demonstrated the amount of content was dependent on the concentration (5, 10, 20, and 30%) of pulp used. Phenolic content recorded was 15.96 ± 1.56, 65.23 ± 1.58, 94.23 ± 2.69, and 123.90 ± 1.33 mg GAE/g with 5, 10, 20, and 30% *Gr.Ex*, respectively The Tukey's test revealed that the phenolic content variation was concentration-dependent. The content in 10, 20, and 30% *Gr.Ex* was significantly higher (*P* < 0.0001) as compared to 5, 10, and 20% *Gr.Ex*, respectively (Figure 2A).

Similarly, total flavonoid contents calculated for all prepared extracts were ranging from 0.1 to 2.5 mg QE/g, and maximal content was discovered with 30% *Gr.Ex*, 2.3 ± 0.15 mg QE/g. The flavonoid content in 10% *Gr.Ex* was significantly higher (*P* = 0.006) as compared to 5% (Figure 2B). The same concentration-dependent rise in anthocyanin content was recorded with significant anthocyanin variation in 10% concentration (*P* = 0.001) as compared to 5% *Gr.Ex*. Similarly, the content fluctuated between 3.20 and 4.12 mg/g for 20 and 30% *Gr.Ex* (Figure 2C).

### Antioxidant Potential

**Figure 3:**
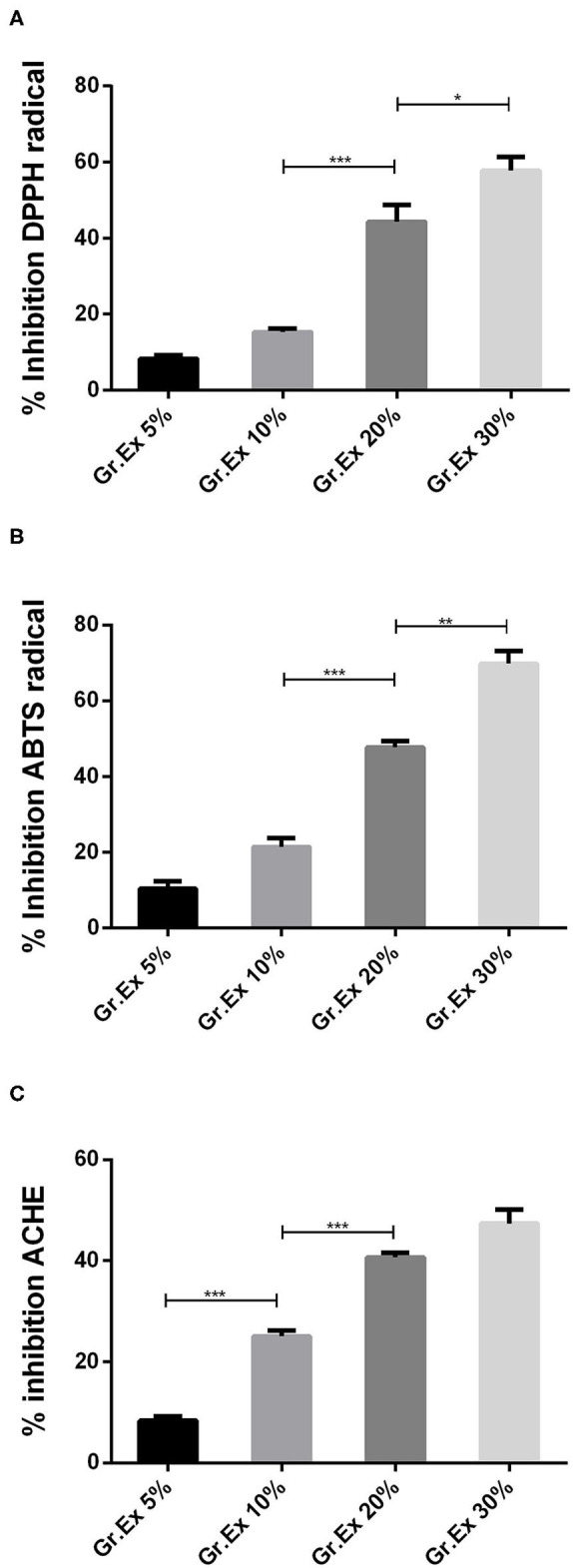
*In vitro* evaluation of 1,1-diphenyl-2-picrylhydrazyl (DPPH) **(A)**, 2,2′-azino-bis-(3-ethylbenzothiazoline-6-sulfonic acid) (ABTS) **(B)**, and acetylcholinesterase (AChE) **(C)** % inhibition capacity of methanolic extracts prepared for different concentrations of fruit juice of *Grewia asiatica*. Data expressed as mean ± SEM (*n* = 3) and evaluated by one-way ANOVA followed by Tukey's test. *P < 0.05, **P < 0.01, ***P < 0.001, comparison between 5 and 10%, 10 and 20%, and 20 and 30% dilutions of *G. asiatica* exudate (*Gr.Ex*).

*In vitro* studies revealed the presence of phenolic, flavonoid, and anthocyanin constituents in the methanolic extract of *G. asiatica* fruit pulp. So, further testing for antioxidant potential was carried out as the plants rich with the abovementioned constituents yield efficient radical scavenging potential. Antioxidant activities were performed through DPPH and ABTS methods, which are among the most reliable *in vitro* methods to estimate the radical hunting capacity of a test substance. In both DPPH and ABTS, the maximum percentage inhibition (>60%) was observed for 30% *Gr.Ex*, with *P* = 0.048 and *P* = 0.001, as compared to lower concentrations, respectively (Figures 3A,B). The outcomes of antioxidant assays found promising to the previously reported studies (6, 49) explain that the fruit of *G. asiatica* was enriched with phytoconstituents that impart this oxidative stress-combating characteristics, which are very essential to deal with a range of diseases.

The AChE inhibitory action by methanol extract of different concentrations of *G. asiatica* fruit pulp demonstrated % inhibition in the range of 8.33–50.66% in a concentration-dependent manner (Figure 3C). The % inhibition of AChE was maximum (50.66%) with 30% *Gr.Ex*, though the activity of the enzyme was inhibited with lower concentrations (5, 10, and 20%) too. This anti-cholinesterase potential is reported to be correlated with the presence of phenolic and flavonoid derivatives (29) in the fruit of *G. asiatica*.

### Anxiolytic Activity

#### Open Field Test

**Figure 4:**
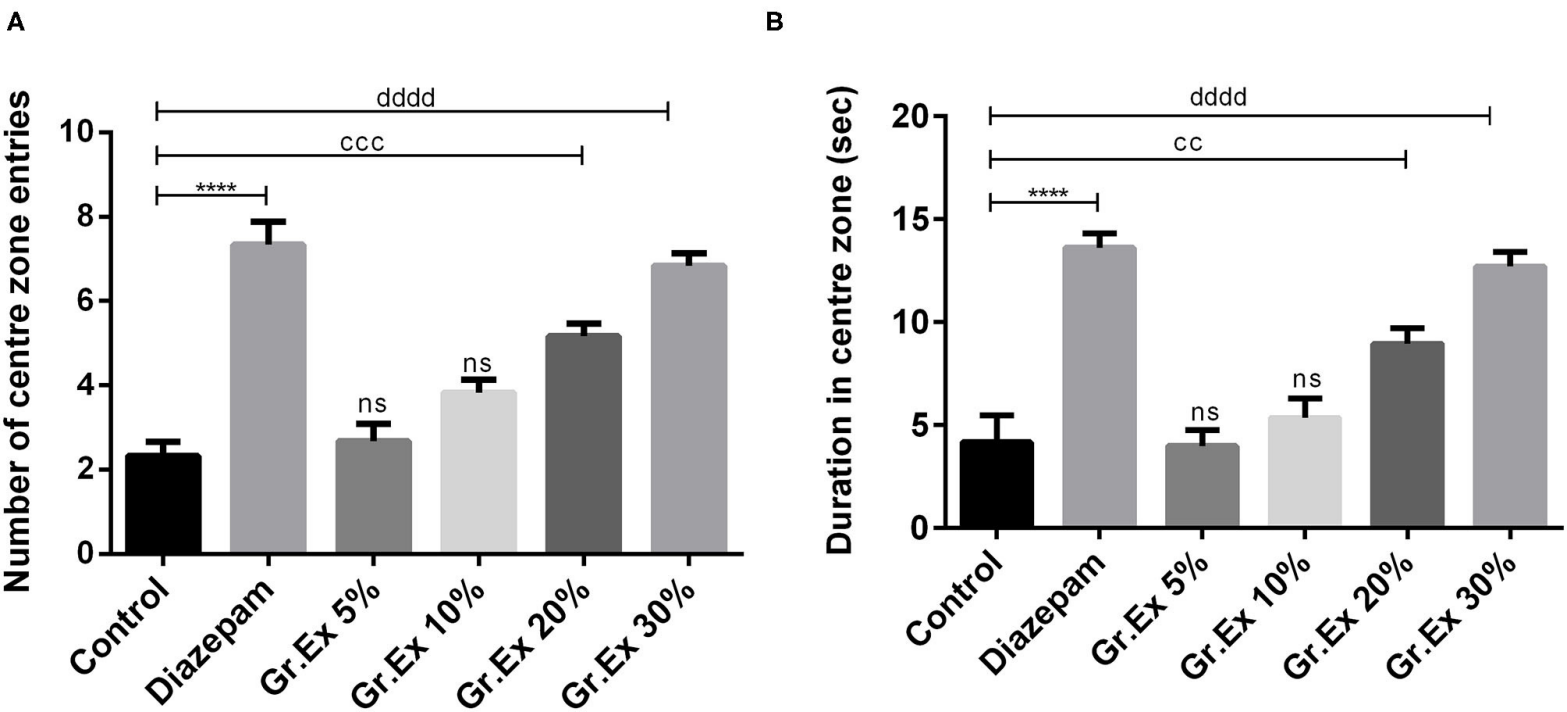
Anxiolytic effect of various dilutions of *Grewia asiatica* exudate (*Gr.Ex*) and diazepam on the **(A)** number of center zone entries and **(B)** duration in the center zone in the open field test. Data expressed as mean ± SEM (*n* = 6) and evaluated by one-way ANOVA followed by Tukey's test. ****P < 0.0001, comparison between control animals and diazepam group. ^cc^*P* < 0.01, ^ccc^*P* < 0.01, comparison between control animals and 20% *Gr.Ex*-treated group. ^dddd^*P* < 0.0001, comparison between control animals and 30% *Gr.Ex*-treated group.

Animals were tested in an open field to evaluate the effect of *Gr.Ex* on anxiety in animals. The exposure to open areas compels animals to stay in peripheries due to anxiety developed in exposed areas as evident from the behavior of animals in the control group. ANOVA revealed a significant difference among all groups for central zone entries [*F*_(5, 30)_ = 30.14, *P* < 0.0001] and time spent in the central area [*F*_(5, 30)_ = 23.74, *P* < 0.0001]. Compared to the control group, animals treated with diazepam (2 mg/kg) demonstrated a significant increase in the number as well as the duration of central zone visits (*P* < 0.0001). Similarly, animals treated with 20 and 30% dilution of *Gr.Ex* exhibited an increased preference for the central area of the field. The number of entries into the central zone was increased in animals treated with 20% (*P* = 0.0002) and 30% (*P* < 0.0001) of *Gr.Ex* (Figure 4A). Likewise, the animals treated with 20 and 30% of *Gr.Ex* spent more time in the central area of field as compared to control animals, with *P* = 0.0071 and *P* < 0.0001, respectively (Figure 4B). However, these results were not significant in animals treated with 5 and 10% dilutions of *Gr.Ex*.

#### Elevated Plus Maze Test

**Figure 5:**
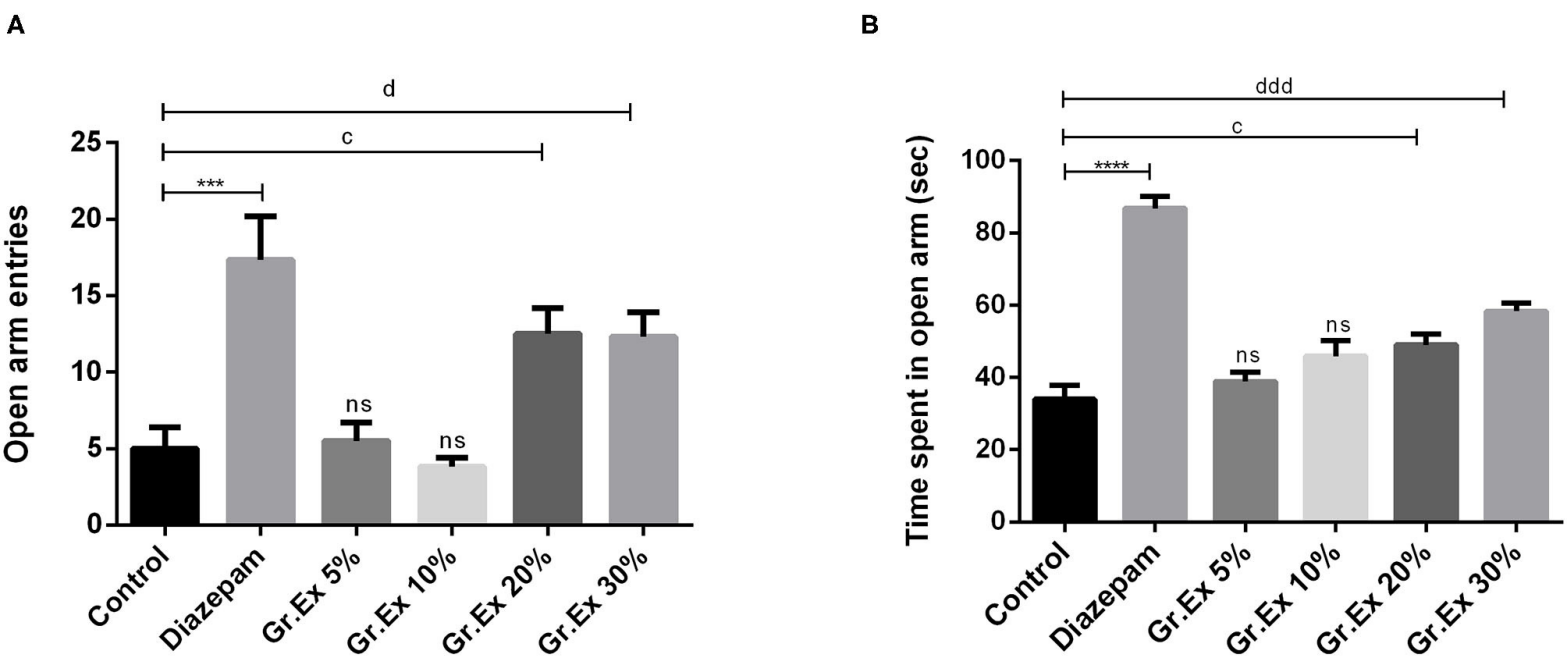
Anxiolytic effect of various dilutions on **(A)** open arm entries and **(B)** time spent in the open arm in the elevated plus maze test. Data expressed as mean ± SEM (*n* = 6) and evaluated by one-way ANOVA followed by Tukey's test. ***P < 0.001, ****P < 0.0001, comparison between control animals and diazepam group. ^c^*P* < 0.05, comparison between control animals and 20% *Grewia asiatica* exudate (*Gr.Ex*)-treated group. ^d^*P* < 0.05, ^ddd^*P* < 0.0001, comparison between control animals and 30% *Gr.Ex*-treated group.

In the EPM test, ANOVA exposed a significant difference between all groups for open arm entries [*F*_(5, 30)_ = 10.27, *P* < 0.0001] and time spent in open arms [*F*_(5, 30)_ = 33.26, *P* < 0.0001]. Rats treated with 20 and 30% dilutions of *Gr.Ex* exhibited significantly increased preference for the open arms, as their entries in open arms of the maze were increased, with*P* < 0.05, as compared to control animals (Figure 5A). Similarly, their duration of stay in the open arms of the maze was also increased in a concentration-dependent manner by 20% (*P* = 0.03) and 30% (*P* = 0.0002) of *Gr.Ex* (Figure 5B). The animals treated with *Gr.Ex* responded the same way as animals treated with diazepam did (*P* < 0.05), revealing the dose-dependent anxiolytic-like potential of *Gr.Ex*.

### Antidepressant Effect

#### Forced Swimming Test

**Figure 6:**
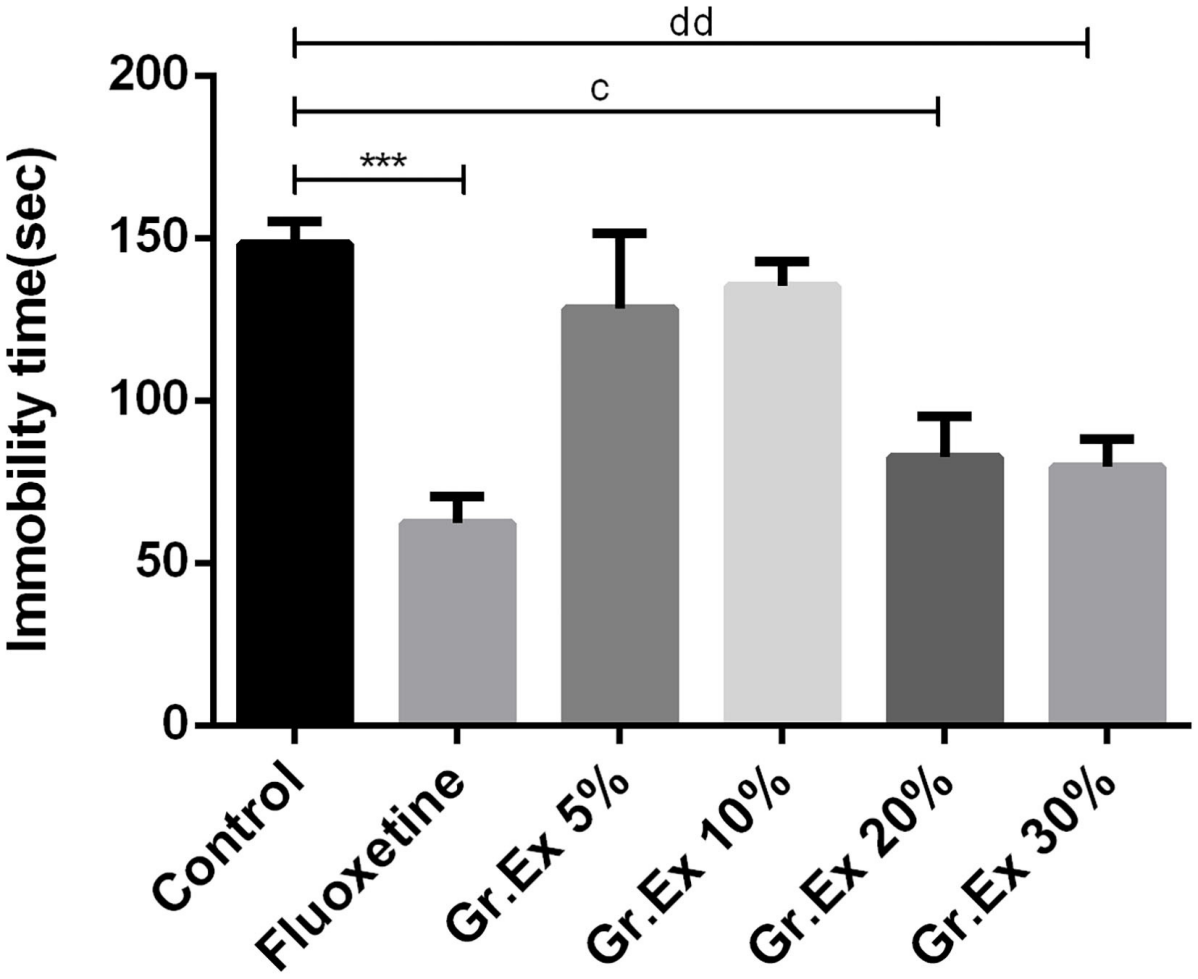
Antidepressant-like effect by various dilutions of *Grewia asiatica* exudate (*Gr.Ex*) on immobility of rats in the forced swimming test. Data expressed as mean ± SEM (*n* = 6) and evaluated by one-way ANOVA followed by Tukey's test. ***P < 0.001, comparison between control animals and fluoxetine group. ^c^*P* < 0.05, comparison between control animals and 20% *Gr.Ex*-treated group. ^dd^*P* < 0.01, comparison between control animals and 30% *Gr.Ex*-treated group.

Outcomes demonstrated noticeable dissimilarity for immobility time [*F*_(5, 30)_ = 8.01, *P* < 0.0001] among all groups, as shown in Figure 6. The immobility time of rats was reduced by standard antidepressant drug, fluoxetine, with *P* = 0.0005. Similarly, the immobility was reduced in rats treated with 20 and 30% dilutions of *Gr.Ex*, with *P* = 0.01 and *P* = 0.006, respectively, as compared to the control animals. However, the results for observed parameter remained unchanged at lower dilutions, i.e., 5 and 10% of *Gr.Ex*.

### Effect on Memory and Spatial Learning

#### Passive Avoidance Test

**Figure 7:**
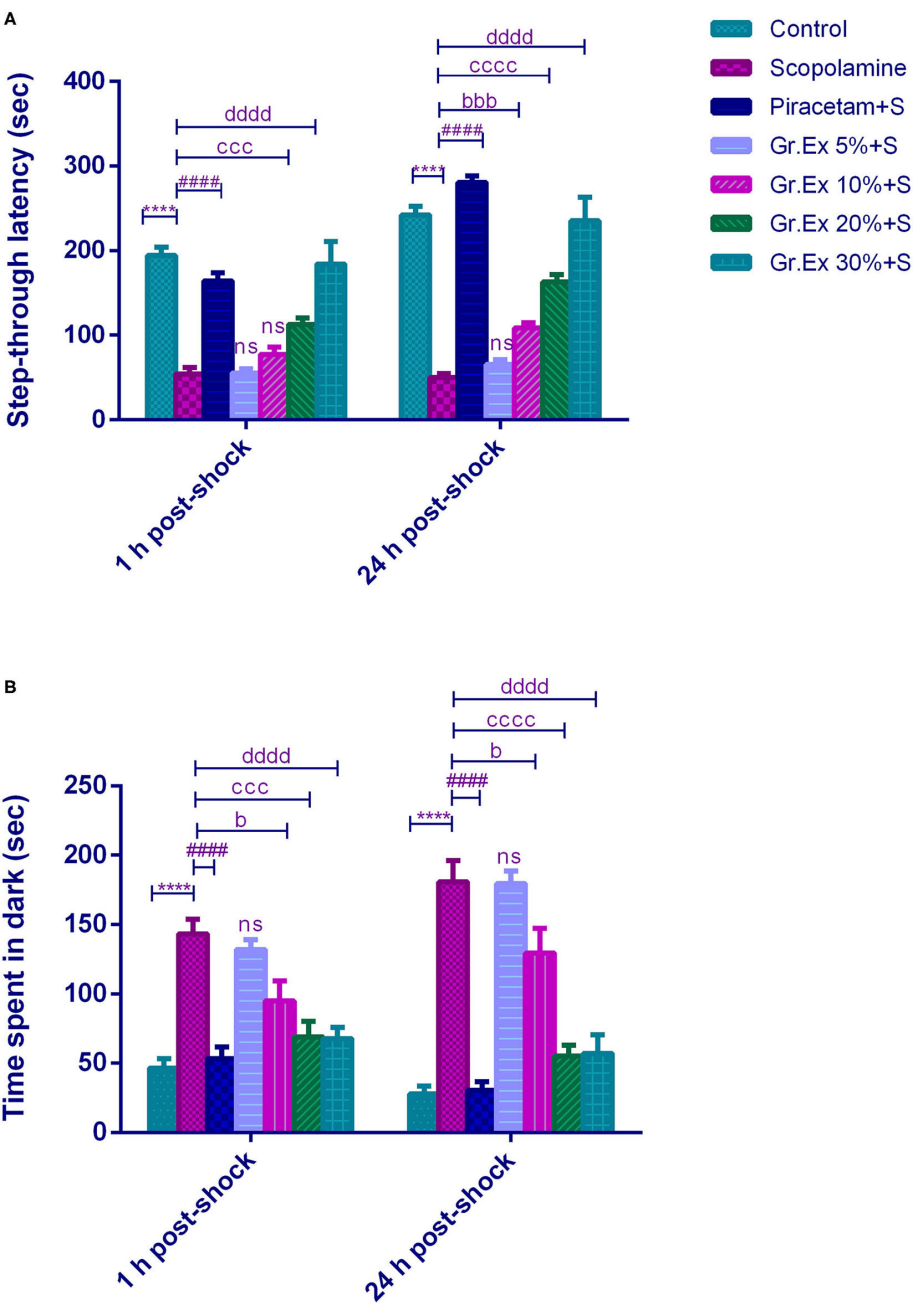
Effect of *Grewia asiatica* exudate (*Gr.Ex*) on **(A)** step-through latencies and **(B)** time spent in the dark in the passive avoidance test. Data expressed as mean ± SEM (*n* = 6) and evaluated by two-way ANOVA followed by Tukey's test. ****P < 0.0001, comparison of control animals with scopolamine-treated group. ^####^*P* < 0.0001, comparison between scopolamine- and piracetam-treated groups. ^b^*P* < 0.05, ^bbb^*P* < 0.001, comparison between scopolamine- and 10% *Gr.Ex*-treated groups. ^ccc^*P* < 0.001, ^cccc^*P* < 0.0001, comparison between scopolamine- and 20% *Gr.Ex*-treated groups. ^dddd^*P* < 0.0001, comparison between scopolamine- and 30% *Gr.Ex*-treated groups.

The PAT revealed the memory-enhancing potential of *Gr.Ex*, as there was improved learning (1 h post-shock trial) and memory retention (24 h post-shock trial). Results demonstrated a significant difference among all groups for step-through latency [*F*_(6, 35)_= 197.0, *P* < 0.0001] and for the animal's stay in the dark compartment [*F*_(6, 70)_ = 47.71, *P* < 0.0001]. The animals treated with scopolamine showed reduced latencies to enter the dark compartment (*P* < 0.0001) as compared to control animals. Piracetam treatment led to a significant increase in latency to enter the darkroom as compared to the scopolamine-treated group (*P* < 0.0001) in both 1 and 24 h post-shock trials. Similarly, scopolamine-induced short latencies were reversed in 1 h post-shock trial by 20 and 30% of *Gr.Ex*, with *P* = 0.0001 and *P* < 0.0001, respectively. The same outcomes were observed in 24 h post-shock trial in which a concentration-dependent effect was observed by 10% (*P* = 0.0001), 20% (*P* < 0.0001), and 30% (*P* < 0.0001) of *Gr.Ex* (Figure 7A).

Likewise, scopolamine-treated amnesic rats spent more time in the dark compartment (*P* < 0.0001) as compared to the control group in both trials. The piracetam-treated animals notably decreased their stay in this shock zone (*P* < 0.0001) as compared to scopolamine-treated rats. The *Gr.Ex* also exhibited improved memory retrieval as animals' stay in the dark compartment was reduced in animals treated with at 10, 20, and 30% *Gr.Ex* dilutions as compared to scopolamine-treated animals, with *P* = 0.036, *P* = 0.0001, and *P* < 0.0001, respectively, in 1 h post-shock trial. The same concentration-dependent outcomes were observed at 24 h where *Gr.Ex*-treated rats spent lesser time in the dark compartment at dilution 10% (*P* = 0.02), 20% (*P* < 0.0001), and 30% (*P* < 0.0001) (Figure 7B).

#### Y-Maze Test

**Figure 8:**
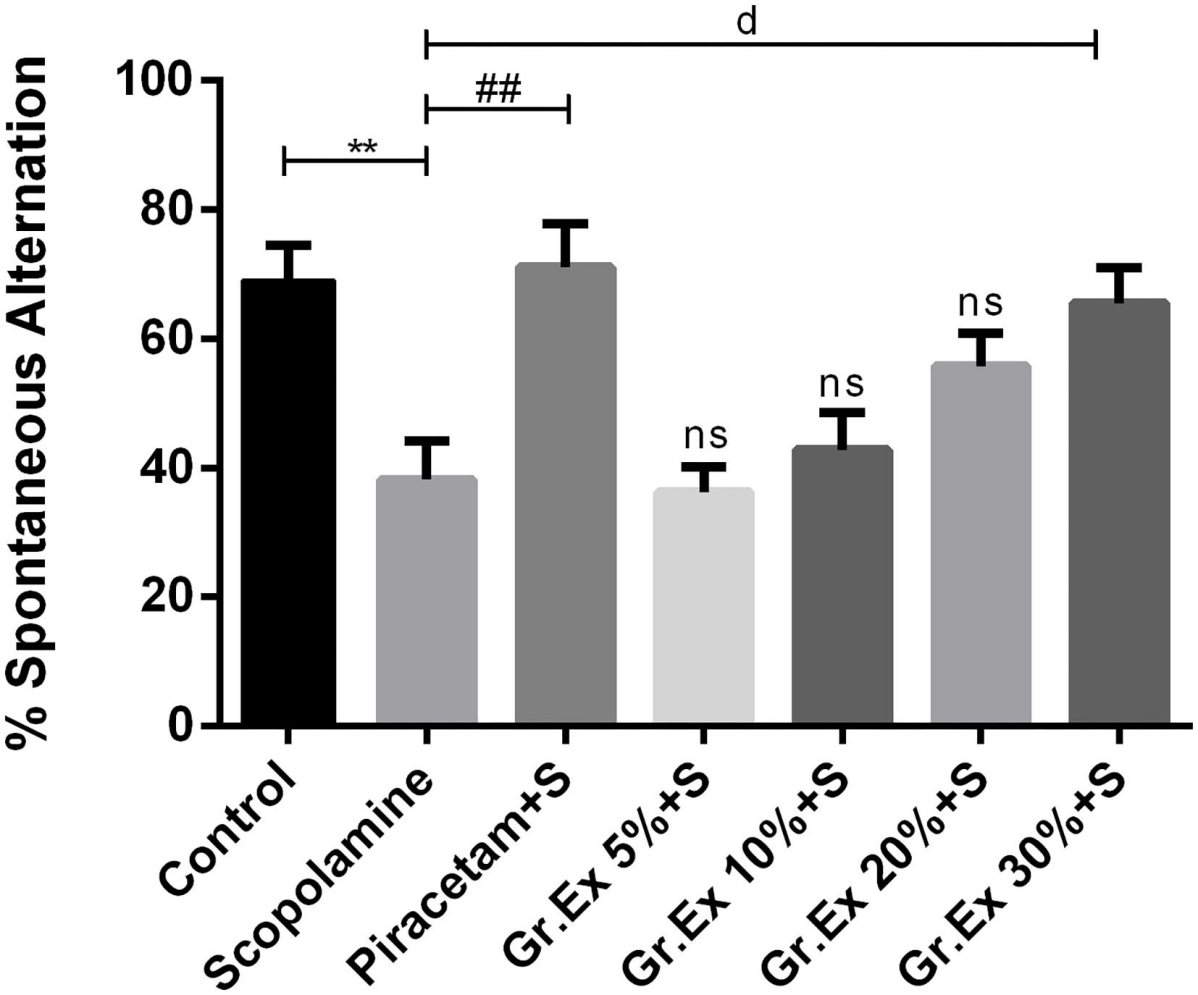
Effect of *Grewia asiatica* exudate (*Gr.Ex*) on spontaneous alternation behavior in the Y-maze task. Data expressed as mean ± SEM (*n* = 6) and evaluated by one-way ANOVA followed by Tukey's test. **P < 0.01, comparison of control animals with scopolamine-treated group. ^##^*P* < 0.01, comparison between scopolamine- and piracetam-treated groups. ^d^*P* < 0.05, comparison between scopolamine and 30% *Gr.Ex*-treated groups.

The Y-maze test was employed to evaluate the effect of the *Gr.Ex* treatment on memory, as increased spontaneous alternation is an indication of enhanced remembrance of already visited arms of the maze (Figure 8). ANOVA disclosed noticeable variations in the gain of a spontaneous alternation score by animals of all treated groups [*F*_(6, 35)_ = 7.359, *P* < 0.0001]. Scopolamine administration induced amnesia in experimental rats, as their score of %SAP was less than that of the control group (*P* = 0.006). Piracetam treatment considerably reversed this memory loss effect of scopolamine (*P* = 0.002). Likewise, the administration of 30% dilution of *Gr.Ex* prominently increased spontaneous alternation behavior in rats (*P* = 0.019), but results were not significant at lower concentrations of *Gr.Ex*.

#### Novel Object Recognition Test

**Figure 9:**
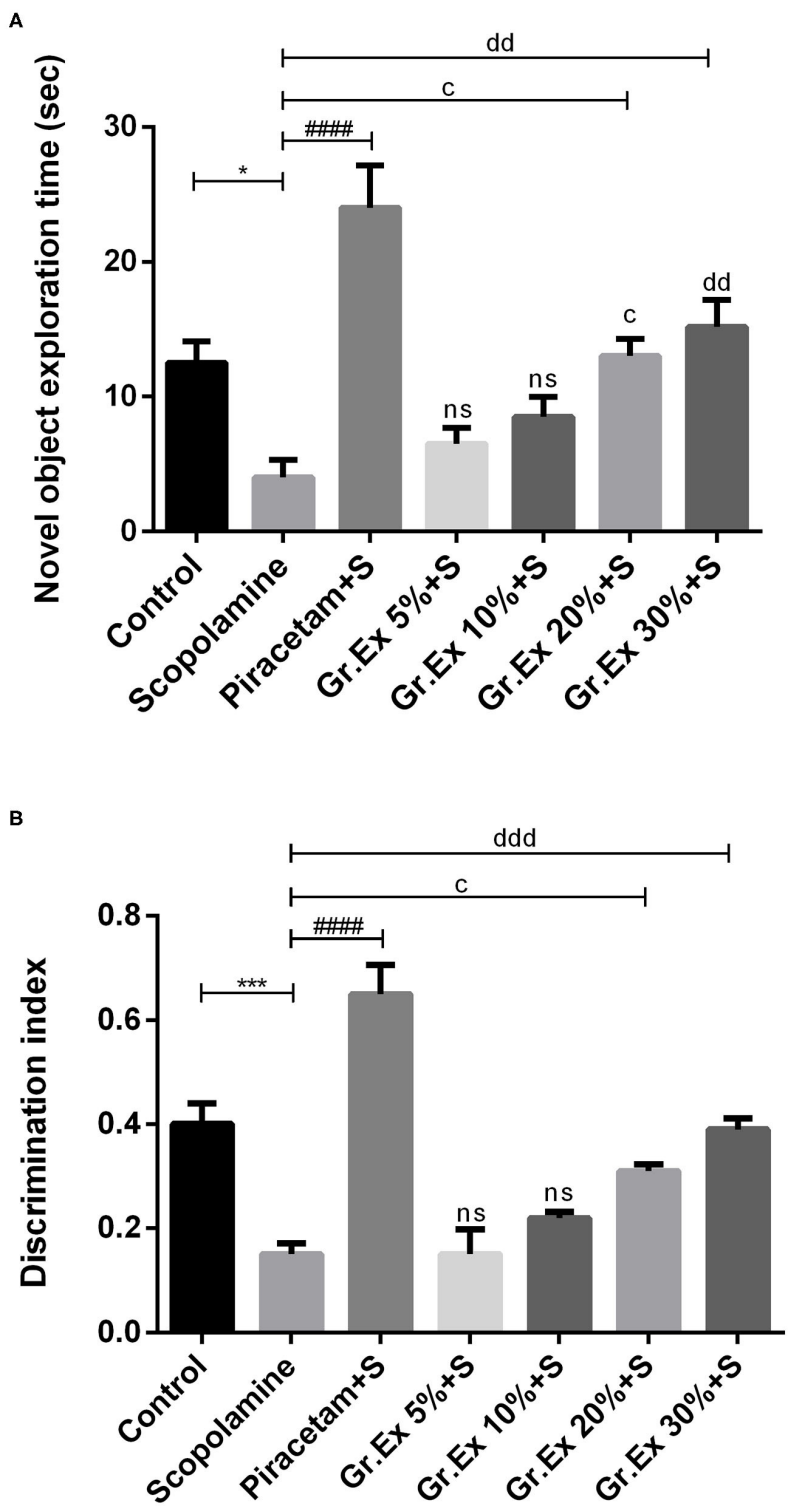
Impact of *Grewia asiatica* exudate (*Gr.Ex*) on **(A)** novel object exploration time and (**B**) discrimination index in the novel object recognition test. Data expressed as mean ± SEM (*n* = 6) and evaluated by one-way ANOVA followed by Tukey's test. *P < 0.05, ***P < 0.001, comparison between control animals and scopolamine-treated group. ^####^*P* < 0.0001, comparison between scopolamine- and piracetam-treated groups. ^c^*P* < 0.05, comparison between scopolamine- and 20% *Gr.Ex*-treated groups. ^dd^*P* < 0.01, ^ddd^*P* < 0.001, comparison between scopolamine- and 30% *Gr.Ex*-treated groups.

Animals treated with *Gr.Ex* 20 and 30% spent more time in the exploration of a novel object as compared to the scopolamine group, with *P* = 0.02 and *P* = 0.002, respectively (Figure 9A). However, scopolamine-induced memory deficit was evident from the behavior of rats who spent less time with the new object compared to the control group (*P* = 0.03). Similarly, data of the discrimination index demonstrated significant differences between all groups during the trial [*F*_(6, 35)_= 26.63, *P* < 0.0001]. Reduced discrimination index exhibited by amnesic rats was reverted by piracetam (*P* < 0.0001) and *Gr.Ex* (*P* = 0.03) (Figure 9B). However, 5 and 10% dilution did not produce significant effects.

#### Morris Water Maze Test

**Figure 10:**
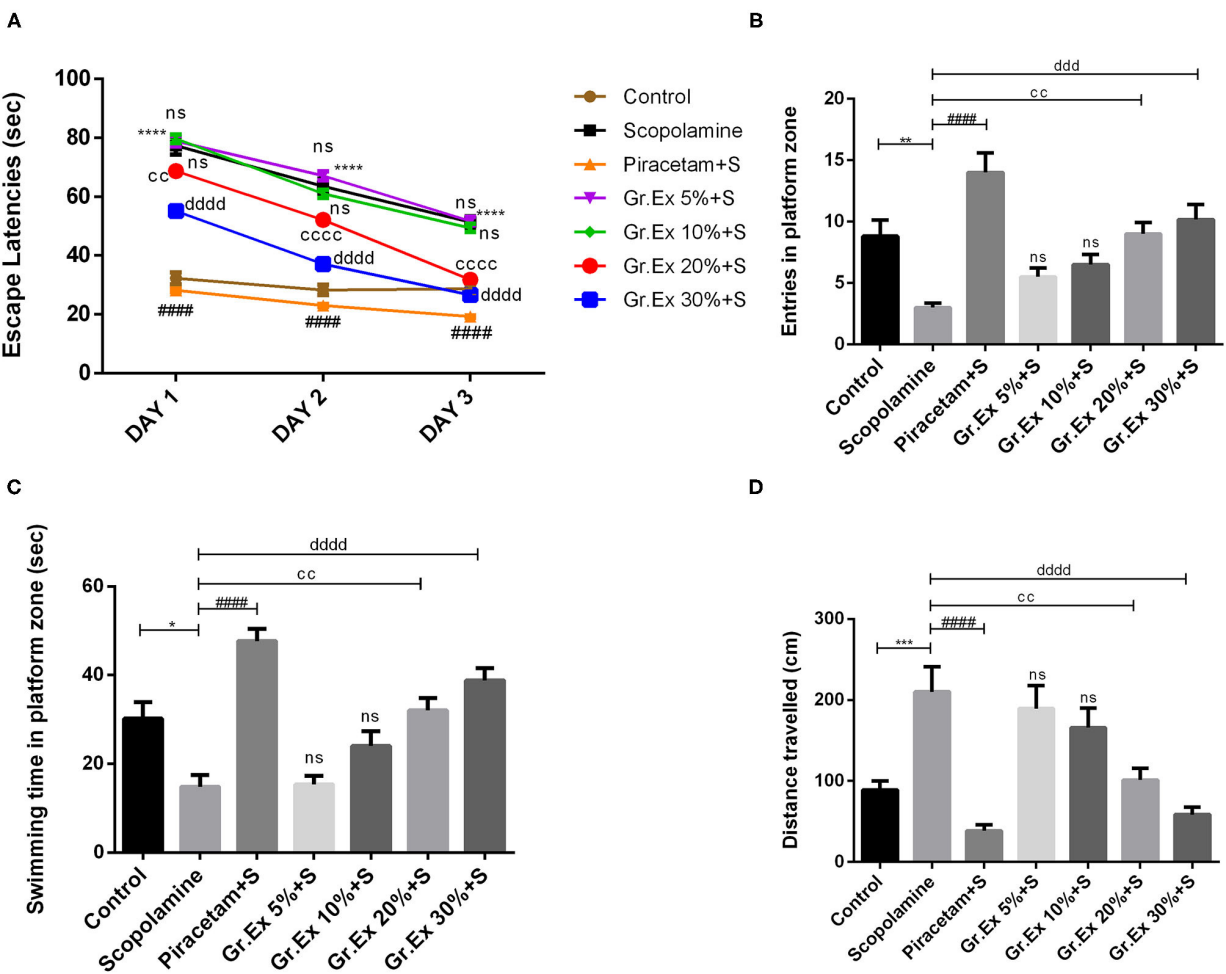
Effect of *Grewia asiatica* exudate (*Gr.Ex*) on **(A)** escape latencies, **(B)** entries in the platform zone, **(C)** swimming time in the platform zone, and **(D)** distance traveled in the Morris water maze test. Data expressed as mean ± SEM (*n* = 6). Escape latencies were evaluated by two-way ANOVA, while one-way ANOVA followed by Tukey's test was used to evaluate other parameters. *P < 0.05, **P < 0.01, ***P < 0.001, ****P < 0.0001, comparison between control animals and scopolamine-treated group. ^####^*P* < 0.0001, comparison between scopolamine- and piracetam-treated groups. ^cc^*P* < 0.01, ^cccc^*P* < 0.0001, comparison between scopolamine- and 20% *Gr.Ex*-treated groups. ^ddd^*P* < 0.001, ^dddd^*P* < 0.0001, comparison between scopolamine- and 30% *Gr.Ex*-treated groups.

The MWM test was employed to evaluate the impact of *Gr.Ex* on scopolamine-induced amnesia, and outcomes were compared with those of piracetam, a nootropic drug. Animals of the control group could locate the hidden platform quickly during training in 3 consecutive days. However, treatment with scopolamine induced prolonged escape latencies in rats (*P* < 0.0001). This amnesic effect of scopolamine was reversed by 20 and 30% *Gr.Ex* in a concentration-dependent manner, with *P* = 0.003 and *P* = 0.0001, respectively, the way piracetam did (*P* < 0.0001) (Figure 10A), and ANOVA revealed significant intergroup differences for escape latencies [*F*_(6, 105)_ = 367.8, *P* < 0.0001]. Results collected on probe day revealed that *Gr.Ex*-treated rats had developed spatial memory, as they showed a preference for the quadrant where the platform was previously situated. As compared to the scopolamine-amnesic rats, the animals treated with 20 and 30% *Gr.Ex* demonstrated an increased number of entries, with *P* = 0.005 and *P* = 0.0003, respectively (Figure 10B). The swimming time in the platform quadrant was also prolonged in a concentration-dependent pattern, showing the better remembrance of platform quadrant in animals exposed to 20% (*P* = 0.003) and 30% (*P* < 0.0001) *Gr.Ex* (Figure 10C). Scopolamine-treated rats showed impaired memory, as they were unable to locate the platform quadrant as compared to the control group (*P* < 0.0001), while piracetam reversed these effects (*P* < 0.0001). Similarly, amnesic rats traveled significantly long distances to reach the target quadrant than the control group (*P* = 0.0007), which was shortened by piracetam (*P* < 0.0001) and *Gr.Ex* (20 and 30%), with *P* = 0.003 (Figure 10D). However, the effects were not prominent in rats exposed to low concentrations (5 and 10%) of *Gr.Ex*.

### Biochemical Analysis of Brain Homogenates

#### Acetylcholinesterase Assay

**Figure 11:**
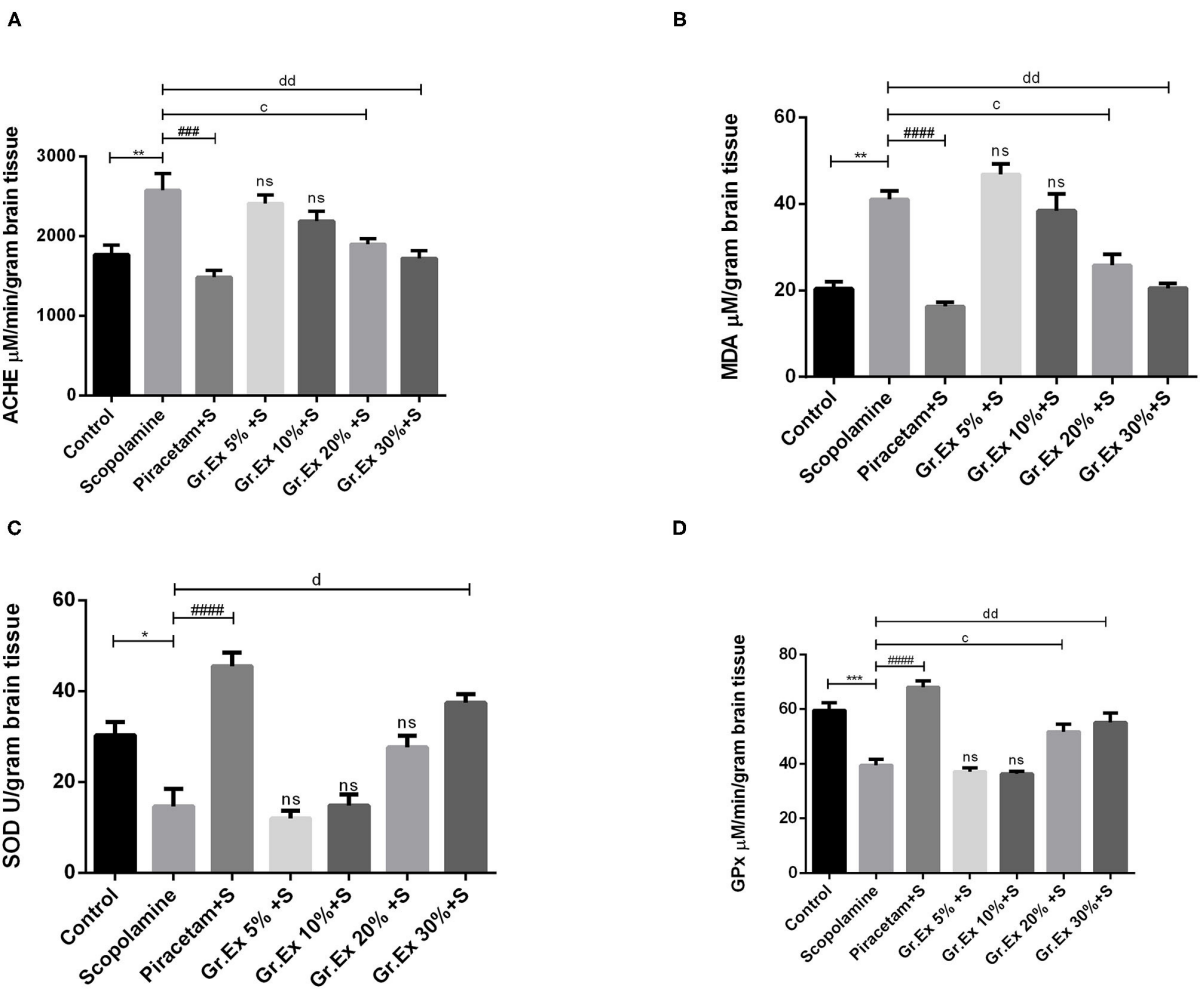
Evaluation of biochemical activity in the brain. Levels of acetylcholinesterase **(A)**, malondialdehyde **(B)**, superoxide dismutase **(C)**, and glutathione peroxidase **(D)**. Data expressed as mean ± SEM (n = 3) and evaluated by one-way ANOVA followed by Tukey's test. *P < 0.05, **P < 0.01, ***P < 0.001, comparison between control animals and scopolamine-treated group. ^###^*P* < 0.001, ^####^*P* < 0.0001, comparison between scopolamine- and piracetam-treated groups. ^c^*P* < 0.05, comparison between scopolamine and 20% *Grewia asiatica* exudate (*Gr.Ex*)-treated groups. ^d^*P* < 0.05, ^dd^*P* < 0.01 comparison between scopolamine- and 30% *Gr.Ex*-treated groups.

A significant difference was revealed by ANOVA among the impact of different treatments on brain AChE level [*F*_(6, 14)_ = 10.26, *P* < 0.001]. Tukey's *post hoc* test unveiled that the administration of scopolamine resulted in significantly increased AChE level than the control group (*P* = 0.005), but piracetam normalized these effects with *P* = 0.0003. Similarly, the elevated AChE levels were reversed by 20 and 30% of *Gr.Ex*, with *P* = 0.023 and *P* = 0.003, respectively (Figure 11A).

#### Malondialdehyde Assay

Biochemical studies of brain homogenate demonstrated significant variation among all groups [*F*_(6, 7)_ = 27.12, *P* < 0.001]. There was a prominently increased MDA level in scopolamine-treated rats as compared to the control group (*P* = 0.004), revealing scopolamine-induced lipid peroxidation in the brain. Administration of *Gr.Ex* reverted these consequences at 20% (*P* = 0.02) and 30% (*P* = 0.004) dilutions the way piracetam did (*P* = 0.001) (Figure 11B).

#### Superoxide Dismutase Assay

SOD is a mitochondrial enzyme that causes the conversion of toxic superoxides to hydrogen peroxide and protects neurons from oxidative damage. Biochemical studies established a noteworthy difference for SOD levels among all treated groups [*F*_(6, 14)_ = 22.35, *P* < 0.0001]. SOD was significantly decreased after scopolamine administration (*P* = 0.01), which may cause oxidative stress and neuronal damage. These unpleasant effects were resolved by piracetam (*P* < 0.0001) and 30% dilution of *Gr.Ex* (*P* = 0.0005) (Figure 11C).

#### Glutathione Peroxidase Assay

GPx is another defense enzyme against oxidative stress in the brain, which is reduced after scopolamine exposure (*P* = 0.0006). The intergroup variation for GPx levels in the brain was significant [*F*_(6, 14)_ = 26.6, *P* < 0.0001]. Piracetam induced improving impact on GPx enzyme level (*P* < 0.0001), and comparable outcomes were also observed with 20% (*P* = 0.03) and 30% (*P* = 0.005) dilutions of *Gr.Ex* (Figure 11D). However, results obtained from animals treated with 5 and 10% dilutions of *Gr.Ex* did not produce any significant effects on the mentioned brain enzymes.

## Discussion

Phenols are secondary metabolites present commonly in a variety of fruits (50). Among their different classes, flavonoids and phenolic acids are categorized as major dietary phenolic compounds (51). Besides this, they are famously known to possess the capacity to combat the oxidative stress built during pathological changes or the natural aging process by reducing the lipid peroxidation in tissues (52). Due to this marvelous potential, they are known to play a role in the protection against a range of diseases including cardiovascular, neurological, and metabolic disorders (51).

In the current investigation, the 70% methanolic extract of different concentrations of *G. asiatica* (5, 10, 20, and 30%) was inquired through *in vitro* testing for the presence of phenols, flavonoids, and anthocyanins, and results revealed the presence of these constituents in the fruit of *G. asiatica*. The correlation of radical scavenging potential with these phytoconstituents was also confirmed through antioxidant assays, and results were following the previous claims (6, 49), providing the hint that the fruit may prove beneficial in managing neurological sufferings (53).

A majority of the world's populace still chooses natural remedies for a range of ailments, as cost and the unpleasant side effects associated with synthetic compounds make it difficult for them to gain adequate treatment. Current researchers are making continuous efforts to examine the curative benefits possessed by nutritional resources (54). Being enriched with a range of phytoconstituents and vitamins, plants own marvelous medicinal properties (55) that may help alleviate the health sufferings of humankind.

*Grewia asiatica* is a nutritional plant that has remarkable medicinal benefits against diabetes, cancer, fever, and aging (6). The fruit exudate of *G. asiatica* has been evaluated in the current study for possible remedial potential in neurological disorders, as the occurrence of anthocyanins, flavonoids, and phenols is confirmed by our *in vitro* analysis. We conducted a series of behavioral tests combined with the biochemical evaluation to see the effectiveness of chronic exposure of *Gr.Ex* on various parameters of anxiety, depression-like behavior, and memory deficit in rodents.

At higher concentrations, *Gr.Ex* produced a prominent anxiolytic effect in rats, as evident from their activity in the behavioral tests for anxiety. Animals administered with 20 and 30% dilutions of *Gr.Ex* were daring enough to visit the central zone and open arms successively in both tests. Diazepam was used as a standard treatment that produces an anxiolytic effect by promoting GABAergic neurotransmission in the brain. As *Gr.Ex* is enriched with valuable phytoconstituents, the anxiolytic-like effect might be linked to the synergistic interaction of its phytochemical constituents. Flavonoids, a family of polyphenolic compounds, are claimed to interact with GABA receptors, thus modulating GABA levels, resulting in neuronal inhibition (56). Our findings are consistent with the previous studies where the ameliorative effect of polyphenol-rich Aronia melanocarpa berries on anxiety was explored by allowing young rats to consume the berry juice for 1 month, and their behavioral analysis demonstrated the positive anxiolytic outcomes (57). Similarly, the rats administered with primary polyphenol in tea (epigallocatechin-3-gallate) for several days revealed a significant anxiolytic effect in open field and light/dark tests (58).

The antidepressant activity was evaluated through an FST in rats, where immobility time was the noted parameter, taking fluoxetine as a positive control that acts as an inhibitor of serotonin reuptake (59). The treatment with different dilutions of *Gr.Ex* exerted a notable antidepressant-like effect in a concentration-dependent manner, as the immobility of rats was decreased after their exposure to a higher concentration of *Gr.Ex*. These characteristics might be existing in *Gr.Ex* due to the presence of flavonoids that may cause inhibition of monoamine oxidases and cause increased levels of monoamines in the brain (59). Besides this, flavonoids are also believed to boost the levels of brain-derived neurotrophic factor (BDNF), an important biomarker of depression, found in the hippocampus; BDNF is a neurotrophin that is known to help in neuronal survival and protection (60). In 2018, a study investigated the association of nutritional polyphenol with symptoms of depression in a cohort of adults, and the outcomes revealed the inverse relationship between both, i.e., the increased dietary flavonoids intake may result in reduced depression-like signs (61). Another study recently reported that naturally-derived polyphenols exert antidepressant-like effects in rats through modulation of the microbiota–gut–brain axis (62). Several lines of criticism are in debate over the recent years with FST or tail suspension test as a tool to judge the antidepressant-like activity (63). According to the researchers, there are possibilities of the uncertainty of the acquired results, animal peculiar behavior in the tank (becomes motionless to get the attention of the experimenter to be pulled out) and terrifying situation for small animals.

Acetylcholine is the major neurotransmitter that acts on widely distributed cholinergic receptors in the brain, and appropriate cholinergic neurotransmission is fundamental for the formation of memory (64). AChE hydrolyzes the acetylcholine, thus diminishing cholinergic neurotransmission. Hence, the assessment of the *in vitro* and *ex vivo* activity of this AChE proves valuable to assess cholinergic activity in the brain and can be further correlated with cognitive function.

The nonselective antimuscarinic drug, scopolamine, passes through the blood–brain barrier (BBB) and mimics the memory impairment symptoms of Alzheimer's disease and aging in rats (65) by impairing cholinergic neuronal transmission (66). In behavioral studies, scopolamine-induced memory deficit was efficiently reverted to normal in rats after *Gr.Ex* (20 and 30%) treatment. Scopolamine-induced shorter latencies to enter the dark compartment were normalized by *Gr.Ex* in the PAT. Animals, treated with fruit exudate, remembered the previously visited arm in Y-maze and formerly explored objects in the novel object recognition test. Similarly, the inability to locate the platform in the MWM test after scopolamine administration was reversed by *Gr.Ex*, as evidenced from shorter escape latencies and preference for quadrant on probe day. Biochemical evaluation of the brain homogenate also demonstrated that scopolamine-induced elevated AChE activity was normalized by *Gr.Ex*, suggesting that inhibition of AChE might be one of the possible mechanisms behind this memory improvement.

Flavonoids present in *Gr.Ex* might inhibit AChE (67), leading to elevated acetylcholine levels in the hippocampus, which in turn may reduce brain pathological changes (68) by downstreaming the neuronal and glial cells responsible for immune responses happening in the brain (69). Flavonoids also bind and activate cAMP response element-binding proteins (CREBs), leading to overexpression of genes influencing memory by strengthening neurotransmission and flow of information (70).

Our findings are in agreement with the previous findings where scientists evaluated the role of a polyphenol-rich extract from grape and blueberry (PEGB) in ameliorating age-related cognitive decline. The mice fed with PEGB for several weeks showed improved spatial learning, memory, and cognition (71, 72), suggesting that polyphenol supplementation can halt the age-related cognitive deficit. In another study, the cognitive function in mice, given coffee polyphenols daily for 5 months, was evaluated using different behavioral tests, and outcomes supported the role of polyphenols in preventing cognitive dysfunction by reducing the amyloid β (Aβ) plaques in the hippocampus (73). Phenol and flavonoids present in *Gr.Ex* could prevent the activation of caspase-3, resulting in antiapoptotic actions and neuronal protection (74). Additionally, they are well-known to promote neuronal proliferation by stimulating the release of neurotrophic factors, thus they may revert the stress-, anxiety-, and aging-impaired adult hippocampus neurogenesis (75).

Scopolamine administration builds oxidative stress in the brain, as evident from reduced SOD and GPx, and increases lipid peroxidation, as marked by elevated MDA (76). Anthocyanins, a major group of polyphenols, are well-known to possess antioxidant potential, which plays a neuroprotective role by reducing intracellular ROS levels. The brain is rich in oxidizable fatty acids and comparatively low antioxidant resistance, which makes the organ more susceptible to oxidative stress. Overproduction of ROS deteriorates cellular components of neurons, leading to apoptosis and damaged learning process (77), as pathogenesis of a range of neurological diseases has a strong association with oxidative stress (78). Furthermore, they also increase neuronal signaling in the hippocampus (79), the part of the brain that processes short-term, long-term, and spatial memory (80). So, these constituents could be credited with the outcomes of the existing study, as many studies have reported their beneficial neuroprotective role (14, 81).

The fruit of *G. asiatica* is also rich in vitamin C (6), which is known to play a crucial role in neuronal maturation and neurotransmission (82). It possesses excellent radical scavenging potential (83) that combats the deteriorating ROS partaking in neurological disorders. Acting as a cofactor in the biosynthesis of carnitine (82), it participates in the production of acetylcholine (84) that improves remembrance by supporting cortical and hippocampal synaptic plasticity. Ascorbic acid is known to enhance the BDNF expression that augments neurogenesis and improves neuronal plasticity essential for learning and memory (85).

## Conclusion

Current findings established the antioxidant, anti-cholinesterase, and neuromodulatory role of phenols, flavonoids, and anthocyanins present in the nutritional fruit of *G. asiatica*. Chronic administration of *Gr.Ex* led to a reduction in anxiety and depression-like behavior as well as reversal of scopolamine-induced cognitive impairment in rats. *Ex vivo* biochemical studies also provided supported the observed outcomes. The effects were more pronounced at higher concentrations of fruit exudate, and reported phytoconstituents might be credited to this phenomenal potential of this fruit. However, molecular mechanisms behind these neuroprotective characteristics need to be further explored and investigated in the future.

## Data Availability Statement

The raw data supporting the conclusions of this article will be made available by the authors, without undue reservation.

## Ethics Statement

All animal studies were performed after availing authorization from the Department of Pharmacology Ethical Committee BZU, Multan (EC/12-PHL-2017 dated 22-05-2017) and were accomplished by following instructions of the Institute of Laboratory Animal Resources (ILAR), Commission on Life Sciences, National Research Council (NRC, 1996).

## Author Contributions

AW, SJ, MR, AM, FA, and II intellectualized the study design, performed the experiment, and analyzed the data and figures. II, MR, NS, HS, MA, FAA, and FA contributed to the assessment of data and statistical analysis. NS, II, and AW performed the biochemical assay. All authors contributed equally in the preparation of the manuscript and approved the final manuscript with consent.

## Conflict of Interest

The authors declare that the research was conducted in the absence of any commercial or financial relationships that could be construed as a potential conflict of interest.
